# Polymer micelle formulation for the proteasome inhibitor drug carfilzomib: Anticancer efficacy and pharmacokinetic studies in mice

**DOI:** 10.1371/journal.pone.0173247

**Published:** 2017-03-08

**Authors:** Ji Eun Park, Se-Eun Chun, Derek Reichel, Jee Sun Min, Su-Chan Lee, Songhee Han, Gongmi Ryoo, Yunseok Oh, Shin-Hyung Park, Heon-Min Ryu, Kyung Bo Kim, Ho-Young Lee, Soo Kyung Bae, Younsoo Bae, Wooin Lee

**Affiliations:** 1 College of Pharmacy and Research Institute of Pharmaceutical Sciences, Seoul National University, Seoul, South Korea; 2 Department of Pharmaceutical Sciences, College of Pharmacy, University of Kentucky, Lexington, Kentucky, United States of America; 3 College of Pharmacy and Integrated Research Institute of Pharmaceutical Sciences, Catholic University of Korea, Bucheon, South Korea; University of South Alabama Mitchell Cancer Institute, UNITED STATES

## Abstract

Carfilzomib (CFZ) is a peptide epoxyketone proteasome inhibitor approved for the treatment of multiple myeloma (MM). Despite the remarkable efficacy of CFZ against MM, the clinical trials in patients with solid cancers yielded rather disappointing results with minimal clinical benefits. Rapid degradation of CFZ *in vivo* and its poor penetration to tumor sites are considered to be major factors limiting its efficacy against solid cancers. We previously reported that polymer micelles (PMs) composed of biodegradable block copolymers poly(ethylene glycol) (PEG) and poly(caprolactone) (PCL) can improve the metabolic stability of CFZ *in vitro*. Here, we prepared the CFZ-loaded PM, PEG-PCL-deoxycholic acid (CFZ-PM) and assessed its *in vivo* anticancer efficacy and pharmacokinetic profiles. Despite *in vitro* metabolic protection of CFZ, CFZ-PM did not display *in vivo* anticancer efficacy in mice bearing human lung cancer xenograft (H460) superior to that of the clinically used cyclodextrin-based CFZ (CFZ-CD) formulation. The plasma pharmacokinetic profiles of CFZ-PM were also comparable to those of CFZ-CD and the residual tumors that persisted in xenograft mice receiving CFZ-PM displayed an incomplete proteasome inhibition. In summary, our results showed that despite its favorable *in vitro* performances, the current CFZ-PM formulation did not improve *in vivo* anticancer efficacy and accessibility of active CFZ to solid cancer tissues over CFZ-CD. Careful consideration of the current results and potential confounding factors may provide valuable insights into the future efforts to validate the potential of CFZ-based therapy for solid cancer and to develop effective CFZ delivery strategies that can be used to treat solid cancers.

## Introduction

The proteasome, a multisubunit protease complex, is an anticancer target validated by remarkable clinical successes of proteasome inhibitor drugs. Since its fast-track FDA approval in 2003, the first-in-class proteasome inhibitor drug bortezomib (Velcade^™^) has become a mainstay of multiple myeloma (MM) therapy, despite drawbacks including severe neurotoxicity caused by off-target interactions with neuronal proteases [1]. In 2012, the second-in-class proteasome inhibitor drug carfilzomib (Kyprolis^™^, CFZ) received an accelerated FDA approval for patients who have relapsed/refractory MM after receiving at least two prior therapies including bortezomib. CFZ in combination with other immunomodulatory agents such as lenalidomide and dexamethasone demonstrated good response profiles and several clinical trials are ongoing for its use as frontline therapies [2–4]. Compared to bortezomib, CFZ is well tolerated with acceptable toxicity profiles and few instances of dose-limiting neurotoxicity, likely due to the selective interactions of its epoxyketone pharmacophore with the proteasome target [5].

Despite the notable benefits with CFZ, there remains much room for improvement. CFZ is practically insoluble in aqueous media and the current formulation contains 60 mg of lyophilized CFZ powder with 3,000 mg of sulfobutylether-β-cyclodextrin (Captisol^®^). Additionally, CFZ is rapidly inactivated *in vivo*; the majority (> 95%) of CFZ is eliminated from systemic circulation within 30 min following intravenous injection due to its peptide backbone cleavage and epoxide hydrolysis [6]. The poor *in vivo* stability and short half-lives of CFZ have been considered major culprits for its lack of efficacy in patients with solid cancers by limiting the access of active drug to proteasome targets within solid tumor tissues [7,8]. Thus, novel drug delivery strategies that can improve solubility, *in vivo* stability of CFZ and the accessibility of active drug to targeted tumor sites may potentially extend its therapeutic benefits in patients with solid cancers.

Polymeric micelles (PMs) composed of amphiphilic block copolymers have gained much attention for their application in drug delivery, especially due to their biocompatibility and utility in improving drug solubility and stability in the biological system and achieving passive tumor targeting, commonly referred to as enhanced permeability and retention (EPR) effect [9,10]. In the case of CFZ, we previously reported that several CFZ-loaded PM formulations composed of biodegradable block copolymers poly(ethylene glycol)-poly(caprolactone) (PEG-PCL) displayed improved metabolic stability and anticancer efficacy profiles *in vitro* [11]. Given these results, the logical next step was to examine whether these *in vitro* improvements achieved by CFZ-loaded PM formulations would be recapitulated *in vivo*.

In this report, we examined the anticancer efficacy and plasma pharmacokinetic (PK) profiles of the CFZ-loaded PM formulation (CFZ-PM, composed of PEG-PCL 5–5.5 KDa with deoxycholic acid added) *in vivo*. Despite our previous results showing *in vitro* metabolic protection with CFZ-PM [11], its *in vivo* performance in terms of anticancer efficacy, plasma PK profiles and proteasome inhibition in residual tumor tissues did not show notable improvements over the cyclodextrin (CD)-based CFZ formulation (CFZ-CD). Careful consideration of these results and potential confounding factors may provide valuable insights into the future efforts to validate the potential of CFZ-based therapy for solid cancer and to develop effective CFZ delivery strategies that can be used to treat solid cancers.

## Materials and methods

### Cell lines and reagents

A human lung adenocarcinoma cell line H460 was obtained from Korean Cell Line Bank (KCLB, Seoul, Korea) and maintained according to the KCLB-recommended culture conditions. CFZ was purchased from LC laboratories (Woburn, VA, USA). Block polymer PEG-PCL with molecular weight 5–5.5 kDa was purchased from Polymer Source (Montreal, QC, Canada). 2-hydroxypropyl-β-cyclodextrin, EDTA, chloropropamide, deoxycholic acid (DCA) and formic acid were purchased from Sigma-Aldrich (St. Louis, MO, USA). The fluorogenic substrate, *N*-Succinyl-Leu-Leu-Val-Tyr-7-amino-4-methylcoumarin (Suc-LLVY-AMC), was purchased from Bachem (Torrance, CA, USA). All solvents for HPLC were obtained from Burdick & Jackson Company (Morristown, NJ, USA).

### Preparation and characterization of CFZ-PM formulation

We prepared the CFZ-PM formulation using PEG-PCL 5–5.5 kDa with DCA through the thin film method as previously described [11]. The particle size distribution and zeta potential values were measured for CFZ-PM or drug-free PM (prepared without CFZ) using an electrophoretic light scattering method (DLS, Zetasizer Nano ZS, Malvern, UK). Critical micelle concentrations (CMCs) of CFZ-PM and empty PM were determined using the pyrene I_3_/ I_1_ method [12]. Briefly, pyrene solution in acetone (2 μM) was added to PM solution in water at varying PM concentrations and left to equilibrate at 37°C overnight. The fluorescence signal intensities of pyrene in the solution were measured at the first (I_1_ at 372 nm) and third (I_3_ at 383 nm) peaks following excitation at 334 nm using a SpectraMax M5 microplate reader in order to determine the encapsulation of pyrene corresponding to micelle formation (SpectraMax M5, Molecular Devices, CA, USA).

### Anticancer efficacy of CFZ-PM in NOD/SCID mice harboring human lung cancer (H460) xenografts

Animal procedure was performed using the protocol approved by the Seoul National University Institutional Animal Care and Use Committee (approval No. SNU-151127-3). NOD/SCID mice were obtained from Japan SLC, Inc. (Hamamatsu, Japan). Briefly, H460 cells (3×10^6^ cells/spot) were subcutaneously injected into the flank of mice (NOD/SCID, 6–7 weeks old, male). After the tumor volume reached 50–150 mm^3^, the mice were randomized into 6 different treatment groups (n = 4–5) as follows; CFZ-PM at the dose of 3 or 6 mg/kg, CFZ-CD (complexed with 20% (v/w) 2-hydroxypropyl-β-cyclodextrin in 10 mM citrate buffer, pH 3) at the dose of 3 or 6 mg/kg, vehicle (10 mM citrate buffer, pH 3), and empty PM. Drug was dosed via tail vein injection (two consecutive days/week for 3 weeks). Tumor growth was assessed by measuring the short and long diameters of the tumor with a caliper and using the following formula: tumor volume (mm^3^) = 0.5 × (short diameter, mm)^2^ × (long diameter, mm). Mice were sacrificed on day 18 (48 h following the last drug treatment) and tumor tissues and whole blood samples were collected for proteasome activity assay and immunoblotting analysis.

### Assessment of proteasome target inhibition/modification in excised xenograft tumor tissues from mice that received drug treatment

In order to assess whether CFZ-PM improved the accessibility of the active drug to tumor tissues, we measured the proteasome target inhibition in excised tumor tissues and whole blood samples from mice that received drug treatment. Tumor tissues and whole blood samples were collected 48 h after the injection of the respective treatments on the last day of the *in vivo* efficacy experiment. The excised tumor tissues were homogenized with passive lysis buffer (Promega, WI, USA) using a hand-held tissue grinder on ice. The homogenates were centrifuged at 3,000g for 20 min at 4°C and the resulting supernatant was used for proteasome activity assay and immunoblotting analysis. The proteasome activity was determined by monitoring the cleavage rate of fluorescent 7-amino-4-methylcoumarine (AMC) from Suc-LLVY-AMC. Briefly, lysates of excised tumor tissues (10 μg of total protein) or whole blood (1 μL) were incubated with Suc-LLVY-AMC (100 μM dissolved in 20 mM Tris-Cl buffer (pH 8.0) containing 500 μM EDTA). Fluorescence signals of liberated AMC were monitored for a period of 60 min using excitation and emission wavelengths of 360 and 460 nm on a SpectraMax M5 microplate reader (Molecular Devices, CA, USA).

Since CFZ irreversibly inhibits the proteasome via covalent modification, the presence of covalently modified catalytic subunit β5 can also be used to assess the extent of the proteasome inhibition. Briefly, tumor tissue lysates (10 μg of total protein) were resolved using 12.5% SDS-PAGE and transferred onto a PVDF membrane (Bio-Rad Laboratories). Membranes were blocked in 5% milk in Tris-buffered saline containing 0.05% Tween-20 (TBST) and probed with the following antibodies; for β5 (dilution 1:1000, Abcam) and β-actin (dilution 1:1000, Cell Signaling). Membranes were washed with TBST and probed with the corresponding secondary antibodies conjugated with horseradish peroxidase. Bound antibodies were visualized using an enhanced chemiluminescence substrate (Thermo Fisher Scientific).

### Assessment of plasma PK profiles of CFZ-PM in mice

Plasma PK studies in mice were carried out following the protocol approved by the Seoul National University Institutional Animal Care and Use Committee (approval No. SNU-160512-5). The CFZ-PM and CFZ-CD were injected via tail vein into ICR mice obtained from Samtako (Gyeonggido, Korea) at the doses of 3 or 6 mg/kg (n = 4–5 per group), respectively. At the pre-determined time points (2, 5, 20, 60, 120, 360, 600 and 1,440 min), whole blood samples were collected from the retro-orbital plexus of the mice using microhematocrit tubes. To minimize blood loss due to sampling, approximately 50 μL of whole blood was drawn at each sampling time and individual mice did not have more than 6 times of blood sampling. Plasma samples (20 μL) separated from whole blood were quenched with acetonitrile (60 μL) containing chlorpropamide (2 μg/mL, an internal standard) and mixed by vortexing for 15 min. After the mixture was centrifuged at 9,000 g for 15 min at 4°C, the concentration of CFZ in the supernatant was measured using an HPLC interfaced with mass spectrometry (Shimadzu LCMS-8050). Briefly, 10 μL of the resulting supernatant was injected and separation of CFZ and chlorpropamide was achieved using a Phenomenex C18 column and the mobile phase composed of H_2_O:acetonitrile (40:60, v/v) containing 0.1% formic acid (flow rate = 0.35 mL/min). CFZ and chlorpropamide were detected in the ESI mode (positive ion mode, CFZ: 720.20→100.15 *m/z*; chlopropamide: 277.05→175.10 *m/z*). The detailed report on analytical conditions and assay validation parameters including accuracy and precision is currently in preparation. PK parameters were calculated using non-compartmental methods (WinNonLin version 5.0.1, Pharsight).

### Statistical analyses

The results were expressed as the mean with standard deviation. Statistical significance between the groups was determined using ANOVA followed by Dunnett’s or Tukey’s *post hoc* test (GraphPad Prism, GraphPad Software Inc., CA, USA). *P* values less than 0.05 were considered to indicate statistical significance.

## Results

### Physicochemical properties of CFZ-PM

The particle size distribution and zeta potential of CFZ-PM and empty drug-free PM were determined using dynamic light scattering. The mean diameters of CFZ-PM and drug-free PM were comparable (56.0 ± 6.1 vs 41.2 ± 5.7 nm) and so were zeta potential values (-0.1 ± 0.4 vs -0.5 ± 0.3 mV) (Table 1). These results indicated that both size distribution and zeta potential were not substantially altered by CFZ drug loading. In addition, particle sizes did not show substantial changes in cell culture media containing fetal bovine serum compared to phosphate-buffered saline (data not shown). These results suggest that PEGylation of micelles may have decreased the tendency for nanoparticles to aggregate. The critical micelle concentration (CMC) values measured by fluorescence spectrophotometry with pyrene were also comparable between drug-free PM and CFZ-PM with 0.18 and 0.14 mg/mL, respectively (Table 1).

**Table 1:** Physicochemical characterization of drug-free and carfilzomib (CFZ) loaded Polymeric Micelles (PM).

Group	Size (nm)	Zeta potential (mV)	CMC (mg/mL)
Drug-free PM	41.2 ± 5.7	-0.5 ± 0.3	0.18
CFZ-PM	56.0 ± 6.1	-1.0 ± 0.4	0.14

Data are shown as means ± S.D. (n = 3). CMC, critical micelle concentration

### *In vivo* anticancer efficacy of CFZ-PM in H460 xenograft mice

**Fig 1:**
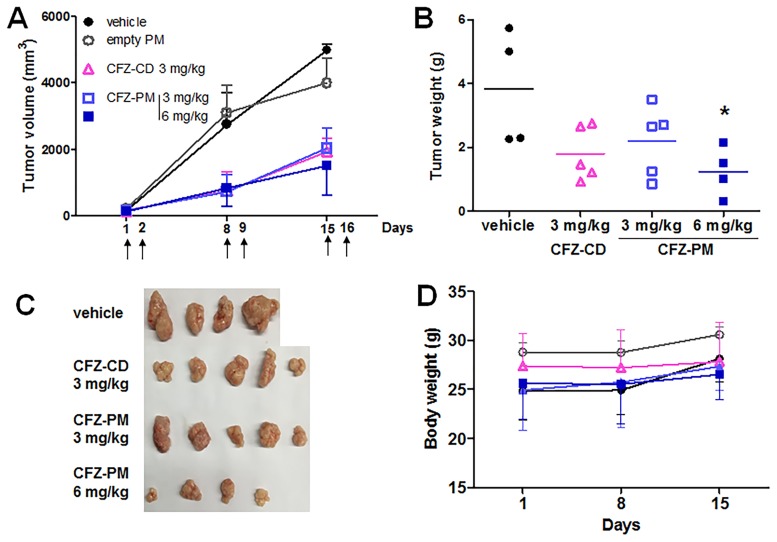
Effects of polymeric micelle formulation containing carfilzomib (CFZ-PM) vs cyclodextrin-based carfilzomib formulation (CFZ-CD) on tumor growth in H460 xenograft mice. NOD/SCID mice harboring H460 xenograft tumors were randomized to 5 different groups and received respective intravenous injections on two consecutive days per week; CFZ-PM at the dose of 3 (□) or 6 (■) mg/kg, CFZ-CD at the dose of 3 (△) mg/kg, vehicle (citrate buffer ●) and empty PM (dissolved in normal saline **○**). The upper arrow symbol (**↑)** indicates the day of drug injection. **(A)** Tumor growth curves. **(B, C)** Weights and images of excised tumor tissues on day 18. **(D)** Body weights. Data are shown as means ± S.D. (n = 4–5). *, *p* < 0.05 vs. vehicle control using ANOVA followed by Dunnett’s *post hoc* test.

The doses and dosing schedules of CFZ-PM and CFZ-CD (3 or 6 mg/kg, intravenous administration on two consecutive days per week) were based on clinically used regimens and available information in the literature [13]. The tumor size was substantially smaller in the groups that received CFZ-PM or CFZ-CD than in the control groups that received empty PM or vehicle only, but no difference was observed between the groups that received CFZ-PM and CFZ-CD at the dose of 3 mg/kg (Fig 1A). We were unable to compare tumor growth suppression of CFZ-PM to that of CFZ-CD at the dose of 6 mg/kg since 4 out of 6 mice that received 6 mg/kg of CFZ-CD died during the treatment period. These results appeared consistent with the literature that reported the maximum tolerated dose of CFZ of 5 mg/kg in mice [14]. The mice that received 6 mg/kg of CFZ-PM survived with no sign of substantial toxicity, at least based on body weight changes (Fig 1D). The tumor growth suppression by CFZ-PM was not however dose-dependent; tumor growth curves for 3 and 6 mg/kg doses overlapped and the weights of excised tumors for both doses were similar (Fig 1B and 1C).

### Proteasome inhibition in post-treatment xenograft tumor tissues and whole blood samples collected from mice that received CFZ-PM or CFZ-CD

**Fig 2:**
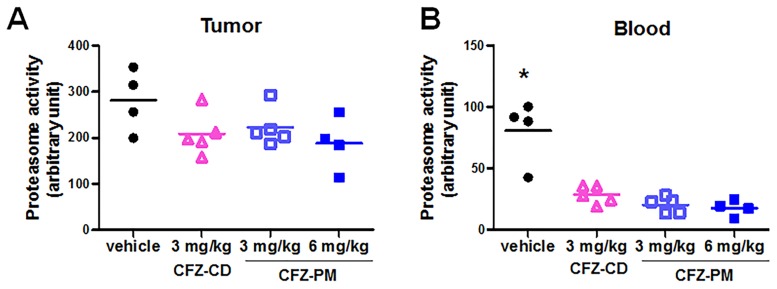
Proteasome activities in the post-treatment tumor tissue lysates (A) and whole blood samples (B) collected from H460 xenograft mice that received the intravenous injections of polymeric micelle formulation containing carfilzomib (CFZ-PM) or cyclodextrin-based carfilzomib formulation (CFZ-CD). The tumor tissues and whole blood samples were collected 48 h after the last injection of the respective treatments. Proteasome activities in tumor tissue lysates or whole blood lysates were assessed by measuring the cleavage rate of the fluorogenic substrate Suc-LLVY-AMC. *, *p* < 0.001 vs. all other groups using ANOVA followed by Dunnett’s *post hoc* test.

In order to probe possible reasons for no enhancement of anticancer efficacy with CFZ-PM over CFZ-CD, we compared the extent of proteasome inhibition in the excised xenograft tumor tissues and whole blood samples collected from the xenograft mice receiving different drug treatments (collected 48 h following the last injection). In the groups that received CFZ-PM (3 or 6 mg/kg) or CFZ-CD (3 mg/kg), the inhibition of the proteasome activity in tumor lysates was modest (and not reaching statistical significance when compared to the control group), with more than 50% of the activity remaining relative to the control group (Fig 2A). In contrast, the proteasome activities in whole blood samples collected 48 h after the last injection of the respective treatments showed an almost complete inhibition in all three tested groups, CFZ-PM (3 or 6 mg/kg) and CFZ-CD (3 mg/kg) (Fig 2B, *p* < 0.001, each treatment different from the control group). Similar results showing an effective and long-lasting inhibition of the proteasome in blood have been reported, especially in the samples collected from patients who received multiple doses of CFZ. In clinical studies that employed repeated dosing schedules with 14- or 28-day cycles (similar to the currently used clinical regimens and that used in our present study), the proteasome activities in whole blood were almost completely inhibited and remained low even in pre-dose samples obtained right before subsequent cycles [15,16]. These findings are also in line with the irreversible, covalent nature of proteasome inhibition by CFZ and the slow proteasome *de novo* biogenesis rates taking at least several days [17–21]. For these reasons, the proteasome activity measured in the lysates of residual xenograft tissues likely represents the proteasome activity from tumor cells where active CFZ did not reach, rather than that recovered following the initial inhibition.

**Fig 3:**
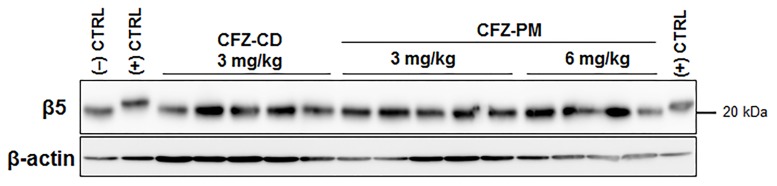
Immunoblotting analyses showing that the proteasome catalytic subunit β5, a primary target of carfilzomib remains unchanged in post-treatment tumor tissue lysates collected from the xenograft mice that received different treatments. (CFZ-CD: cyclodextrin-based carfilzomib formulation; CFZ-PM: polymeric micelle formulation containing carfilzomib) The tumor tissues were collected 48 h after the last injection of the respective treatments.

In addition to measuring post-treatment proteasome activities, we also probed the extent of CFZ-induced, covalent modification of the major proteasome catalytic subunit β5. This assay takes advantage of the altered electrophoretic mobility of the covalently modified β5 protein by CFZ as previously described [22]. The band of the positive control (tumor lysates incubated with 50 nM CFZ for 2 h *in vitro*) showed a mobility shift compared to the negative control (tumor lysates incubated with the vehicle DMSO for 2 h) (Fig 3). No detectable β5 band shift was observed in the post-treatment tumor lysates collected 48 h after the last drug treatment in either CFZ-PM or CFZ-CD groups. Altogether, these results suggest that CFZ-PM did not enhance the access of active CFZ to cancer cells in xenograft tumors and the extent of proteasome inhibition *in vivo*.

### Comparison of plasma PK profiles of CFZ-PM and CFZ-CD in ICR mice

**Fig 4:**
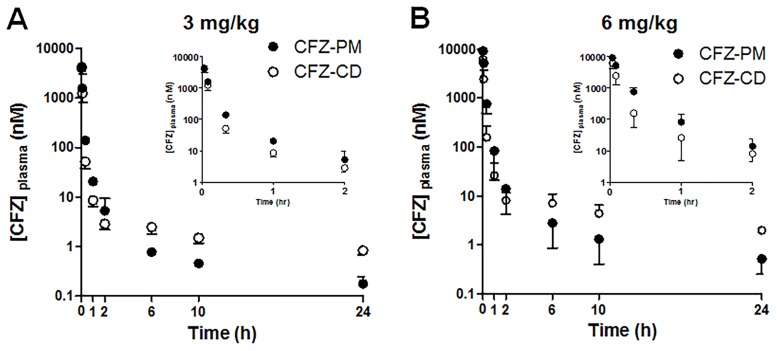
Plasma concentration-time profiles of carfilzomib after the intravenous administration of polymeric micelle formulation containing carfilzomib (CFZ-PM) or cyclodextrin-based carfilzomib formulation (CFZ-CD) to mice (A, 3 mg/kg and B, 6 mg/kg). Data are shown as means ± S.D. (n = 4–5). The inset figures show the plasma concentration-time profiles up to 2 h.

Given that CFZ-PM did not suppress the tumor growth rate more effectively than CFZ-CD (Fig 2), we examined whether the plasma PK profiles differ between CFZ-PM and CFZ-CD. Following the intravenous injection of CFZ-PM or CFZ-CD to ICR mice, the plasma concentration-time curves for both groups displayed a rapid decline. During early time points (up to 2 h), the mice that received CFZ-PM (either 3 or 6 mg/kg) showed higher drug concentrations than those that received CFZ-CD (Fig 4). However, this trend was reversed in later time points (6, 10 and 24 h after injection); the mice that received CFZ-PM injection (3 or 6 mg/kg) showed lower CFZ concentrations in plasma than those that received CFZ-CD.

For detailed comparison, the PK parameters were obtained using non-compartmental methods (Table 2). As expected from the rapid initial decline in the plasma concentration-time curves, the AUC_0-2h_ values accounted for approximately 96.7% and 97.0% of the AUC_INF_ values in the CFZ-PM groups at the doses of 3 and 6 mg/kg, respectively. In the case of the CFZ-CD groups, the AUC_0-2h_ values accounted for approximately 91.4% and 88.1% of the AUC_INF_ values at the doses of 3 and 6 mg/kg, respectively. The CL values of CFZ in all four groups exceeded the average hepatic blood flow in mice, suggesting substantial extrahepatic metabolism of CFZ. These results are in line with the previous reports in rats and humans [6,8]. The systemic exposure of CFZ-PM and CFZ-CD at the dose of 3 mg/kg appeared to be comparable based on the similar AUC_INF_ and CL values between the two groups. When the AUC_INF_ values of 3 and 6 mg/kg doses were compared, the CFZ-PM group displayed approximately 3-fold increases (39.9 ± 0.8 vs 121.8 ± 27.4) while the CFZ-CD group displayed approximately 1.7-fold increases (36.4 ± 8.0 vs 62.6 ± 22.9). These differences in the AUC_INF_ values led to a slower CFZ clearance in the CFZ-PM group at the dose of 6 mg/kg than the other groups (*p* < 0.05). When the half-lives were compared during the initial decline phase (up to 2 h), the t_1/2, initial_ values were shorter than 30 min and comparable between CFZ-PM and CFZ-CD groups. These results are consistent with the previously reported half-lives in rats and humans [6,8]. The half-lives from the terminal phase (t_1/2, terminal_) were in the ranges of several hours and these results may reflect differing experimental conditions including the assay sensitivity (our analytical assays detected much lower CFZ concentrations of near 1 nM than the previous reports [6,8]). The values of MRT and V_ss_ were much decreased in the mice receiving CFZ-PM compared to those receiving CFZ-CD. It should be however noted that our analytical assays measured the total CFZ concentrations in plasma (both micelle-entrapped CFZ and the free CFZ released from the micelles), so the PK parameters associated with biodistribution may need to be interpreted with caution.

**Table 2:** Pharmacokinetic parameters of carfilzomib after the intravenous administration of polymeric micelle formulation containing carfilzomib (CFZ-PM) and cyclodextrin-based carfilzomib formulation (CFZ-CD) to ICR mice.

Pharmacokinetic Parameters	CFZ-PM		CFZ-CD	
	3 mg/kg	6 mg/kg	3 mg/kg	6 mg/kg
AUC_0-2h_ (min*nmol/mL)	38.6 ± 1.2	118.1 ± 25.3	33.3 ± 7.7	55.2 ± 21.1
AUC_0-24h_ (min*nmol/mL)	39.8 ± 0.8	121.4 ± 27.2	35.4 ± 8.0	58.8 ± 21.8
AUC_INF_ (min*nmol/mL)	39.9 ± 0.8	121.8 ± 27.4	36.4 ± 8.0	62.6 ± 22.9
CL (mL/min/kg)	105 ± 2	71 ± 15	119 ± 29	152 ± 71*
C_0_ (nmol/mL)	8,490 ± 1,550	11,990 ± 1,150	9,940 ±4,860	11,760 ± 3,440
t_1/2, initial_ (min)	21.2 ± 5.0	17.4 ± 1.1	24.8 ± 1.0	22.4 ± 7.3
t_1/2, terminal_ (h)	6.1 ± 1.4*	8.5 ± 1.7	13.1 ± 3.5	11.1 ± 4.8
V_ss_ (L/kg)	2.19 ± 0.40	1.62 ± 0.07	13.42 ± 6.58*	20.14 ± 10.38**
MRT (min)	20.9 ± 3.6	23.9 ± 5.7	110.9 ± 46.3*	136.2 ± 70.5**

Data are shown as means ± S.D. (n = 4–5). AUC_0-2h_, Area under the plasma concentration-time curve (AUC) from time 0 to 2 h; AUC_0-24h_, AUC from time 0 to 24 h; AUC_INF_, AUC extrapolated to infinity; CL, clearance; C_0_, extrapolated plasma concentration at 0 h; t_1/2, initial_, half-life from initial decline phase (0 to 2 h); t_1/2, terminal_, terminal half-life (0 to 24 h); V_ss_, volume of distribution at steady state; MRT, mean residence time.

*, *p* < 0.05

**, *p* < 0.01 vs. all other groups using ANOVA followed by Tukey’s *post hoc* test.

## Discussion

The proteasome is well accepted as a critical player in several traditional hallmarks of cancer, defined by Hanahan and Weinberg [23]. Proteotoxic stress triggered by imbalances in protein homeostasis has been recently annotated as another hallmark of cancer [24]. In this regard, CFZ with improved efficacy and safety profiles merits further investigations to extend its therapeutic utility beyond MM. Recent reports increasingly suggested that certain solid cancers render proteasome addiction as vulnerability, thereby a potential target for therapeutic interventions. Using genome-wide siRNA screening, the knockdown of proteasome genes was found to cause lethality in basal-like triple-negative breast cancer cells [25]. This particular study examined the effectiveness of bortezomib administered via different dosing routes in suppressing *in vivo* tumor growth and metastasis. Only intratumoral injection, but neither intraperitoneal nor intravenous injection, displayed an efficient proteasome inhibition associated with enhanced anticancer efficacy [25]. An early report with bortezomib also indicated that intratumoral injection of the drug leads to an effective proteasome inhibition and growth suppression in mice harboring prostate cancer xenografts [26]. Altogether, these findings provide an impetus to develop novel delivery strategies that can effectively target proteasomes in solid cancer cells and harness the potential of CFZ-based therapy for solid cancer patients.

Previously, we developed several CFZ-loaded PM formulations displaying improved metabolic stability and anticancer efficacy *in vitro* [11]. In the current study, we investigated *in vivo* anticancer efficacy and plasma PK profiles of CFZ-PM (CFZ-loaded PEG-PCL 5–5.5 KDa with DCA) in mice. In the human lung cancer xenograft model, CFZ-PM did not show substantial improvements in the anticancer efficacy and proteasome inhibition at the tumor sites over CFZ-CD (Figs 2 and 3). In addition, the plasma PK profiles of CFZ-PM were for the most part comparable to those of CFZ-CD at the dose of 3 mg/kg except showing slightly higher drug concentrations at early time points (Fig 4). Our results indicated an incomplete proteasome inhibition in the post-treatment tumor tissues collected from H460 xenograft mice 48 h after the last injection of CFZ-PM (Figs 2 and 3). In further probing possible reasons for the lack of improvements with CFZ-PM over CFZ-CD, it would be important to examine whether the xenograft model employed in the current study allowed for sufficient passive targeting effect. Although the particle size distribution of CFZ-PM (56.0 ± 6.1 nm, Table 1) is sufficiently small to pass through the pore size of vascular membranes (60 ~ 100 nm) [27,28], our results suggest that CFZ-PM was not more effective in providing the access of active CFZ into tumor sites than CFZ-CD. These results may be reflective of potential confounding factors limiting the access of active drug to cancer cells, such as heterogeneity in tumor and its surrounding vasculature, abnormal tumor blood vessels and high interstitial fluid pressure [29,30]. To overcome such obstacles, various pharmacological and physical strategies including focal radiation and sonoporation have been exploited in the field [31–33]. It would be important to obtain more detailed biodistribution data and to consider combining approaches to improve tumoral penetration of active CFZ in future investigations.

The extent and rate of CFZ release from CFZ-PM *in vivo* may also be a factor influencing anticancer efficacy. The plasma PK profiles of CFZ-PM after a single intravenous administration displayed higher drug concentrations during the initial phase (up to 2 h) than those of CFZ-CD at the dose levels of both 3 and 6 mg/kg (Fig 4). When the plasma PK parameters were compared, mice that received CFZ-PM (6 mg/kg) displayed a greater systemic exposure and a slower CFZ clearance than those that received CFZ-CD (6 mg/kg) (Table 2). However, the systemic toxicity appeared to be more severe in xenograft mice treated with CFZ-CD (6 mg/kg) than those treated with CFZ-PM (6 mg/kg); 4 out of 6 mice receiving 6 mg/kg repeated doses of CFZ-CD died. Although these results need to be interpreted with caution due to the small sample size, they may suggest that CFZ-PM may have the potential to increase maximum tolerated dose levels and to influence CFZ release kinetics and biodistribution profiles compared to CFZ-CD. Of note, the stability of micelles is another important consideration for CFZ-PM formulation. Given the relatively low drug loading efficiency (2.3%) of the current CFZ-PM [11], the initial micelle concentrations (upon immediate dilution of the CFZ-PM 3 mg/kg dose in an average mouse blood volume of approximately 2 mL) are estimated to be well above the measured CMC value (0.14 mg/mL, Table 1). However, as polymers are cleared from blood, micelle concentration will decrease and micelles could degrade and release CFZ. Thus, it might be necessary to explore polymer-based nanoparticles stabilized with various structural/functional modifications [10,27]. For the docetaxel-loaded PM formulation composed of the same block copolymer, PEG-*b*-PCL, the CMC value of 0.02 mg/mL was reported in *in vitro* conditions [34]. Thus it may be feasible to lower CMC values for CFZ-loaded PM formulations by carefully optimizing various factors (e.g., drug-to-polymer ratios, addition of excipients stabilizing hydrophobic cores) using the current block copolymer or by using different types of block copolymers.

In summary, the results in this study showed that the current CFZ-PM does not enhance anti-cancer efficacy *in vivo*. Careful consideration of these results and confounding factors may provide valuable insights into the future efforts to validate and harness the potential of CFZ-based therapy for solid cancer by developing effective CFZ delivery strategies.
